# Neurodevelopmental outcome of very preterm infants with gastrointestinal tract perforations does not differ compared to controls

**DOI:** 10.1007/s00508-021-01886-z

**Published:** 2021-06-10

**Authors:** Michael F. Moser, Irina J. Müller, Johannes Schalamon, Bernhard Resch

**Affiliations:** 1 Research Unit for Neonatal Infectious Diseases and Epidemiology, Medical University of Graz, Auenbruggerplatz 34/2, 8036 Graz, Austria; 2 Division of Pediatric Surgery, Department of Pediatric and Adolescent Surgery, Medical University of Graz, Auenbruggerplatz 34/1, 8036 Graz, Austria; 3 Division of Neonatology, Department of Pediatrics and Adolescent Medicine, Medical University of Graz, Auenbruggerplatz 34/2, 8036 Graz, Austria

**Keywords:** Meconium obstruction of prematurity, Spontaneous intestinal perforation, Necrotizing enterocolitis, Volvulus, Ileus, Follow-up

## Abstract

**Purpose:**

To evaluate gastrointestinal tract (GIT) perforations in very low birth weight infants and the effects on neurodevelopmental outcome.

**Methods:**

Between 2000 and 2017 all cases with GIT perforation were analyzed regarding causes, associated morbidities and neurodevelopmental outcome and compared with matched (gestational age, birth weight, gender, year of birth) by 1:2 controls.

**Results:**

The incidence of GIT perforation was 2.0% (*n* = 38/1878). Diagnoses associated with GIT were meconium obstruction of prematurity (MOP,*n* = 19/50%), spontaneous intestinal perforation (SIP, *n* = 7/18%), necrotizing enterocolitis (NEC, *n* = 6/16%), iatrogenic perforation (*n* = 3/8%), volvulus (*n* = 2/5%) and meconium ileus (*n* = 1/3%). The NEC-associated perforations occurred later compared to those associated with MOP and SIP (median 8 days and 6 days vs. 17 days, *p* = 0.001 and 0.023, respectively) and main localization was the terminal ileum (84%). Cases had higher rates of late onset sepsis (55% vs. 24%, *p* = 0.003), longer duration of mechanical ventilation (median 30 days vs 18 days, *p* = 0.013) and longer stays at the hospital (median 122 days vs 83 days, *p* < 0.001); mortality rates did not differ. The 2‑year neurodevelopment follow-up revealed no differences between groups (normal development 49% vs. 40%).

**Conclusion:**

Despite increased morbidities preterm infants with GIT perforation did not have a higher mortality rate and groups did not differ regarding neurodevelopmental outcome at the corrected for prematurity age of 2 years.

## Introduction

Perforations of the gastrointestinal tract (GIT) are a severe and life-threatening complication for preterm infants [1]. Necrotizing enterocolitis (NEC), spontaneous intestinal perforation (SIP) and obstructive complications, such as delayed meconium passage (meconium obstruction of prematurity) or volvulus present common causes for this neonatal emergency [2–5].

In some studies, mortality was reported to be higher in preterm infants with GIT perforations than in those without [6, 7]. Furthermore, among survivors infants with perforation have shown a worse neurodevelopment outcome than those without [6, 7]; however, other studies showed no significant differences in mortality and neurodevelopment between infants with perforation and those without. Researchers suggested that neurodevelopmental deficits and mortality are caused by prematurity in general, rather than GIT perforation in particular [8].

At the Division of Neonatology of the Medical University of Graz, we also experienced neonatal GIT perforations and their consequences [9] but cases of the recent past have not been part of an academic study yet. We hypothesized that preterm infants with GIT perforations had more morbidities, a higher mortality rate and a worse neurodevelopmental outcome at the age of 2 years.

Thus, we analyzed cases of preterm infants(≤ 32 + 6 weeks of gestational age) with GIT perforations and assessed the impact of GIT perforation on mortality and neurodevelopmental outcome.

## Material and methods

The study was approved by the local ethics committee of the Medical University of Graz (30-152 ex 17/18).

### Study design and patients

This was a single-center retrospective matched case-control chart review study, which analyzed cases of gastrointestinal tract (GIT) perforations and determined the neurodevelopmental outcome at the age of 2 years corrected for prematurity by means of a 1:2 matched case-control study according to the Strengthening the Reporting of Observational Studies in Epidemiology (STROBE) criteria (supplementary file). All inborn preterm infants (gestational age of ≤ 32 + 6 weeks) suffering from a GIT perforation in the neonatal period between 1 January 2000 and 31 December 2017 were included. Matching criteria included the year of birth, gender, gestational age (±1 week) as well as the birth weight (±200 g).

### Data collection

The perinatal and neonatal data as well as data concerning the perforation and the follow-up including neurodevelopmental outcome were evaluated for all patients. The data were retrieved from the local electronic data management system called openMedocs© (Styrian medical documentation and communication network, Krankenanstaltengesellschaft-KAGES, Styria, Austria) and collected using Microsoft Excel© (Richmond, WA, USA). Perinatal data included date of birth, gender, gestational age (weeks), birthweight (g), small for gestational age (SGA, birth weight < 10th percentile), mode of delivery, multiple pregnancy (twins, triplets), chorioamnionitis, maternal age, Apgar scores at 1 min and 5 min and umbilical artery pH. Neonatal data included the need for mechanical ventilation (days), duration of hospitalization (days), early onset sepsis (EOS), respiratory distress syndrome (RDS), RDS grading (1–4), intraventricular hemorrhage (IVH), IVH grading (mild and severe), periventricular echo densities (PVE), cystic periventricular leukomalacia (PVL), neonatal seizures, late onset sepsis (LOS), bronchopulmonary dysplasia (BPD), retinopathy of prematurity (ROP) as well as ROP grading (1–5) and delayed meconium passage (meconium obstruction of prematurity). The latter was defined as meconium defecation at more than 48 h of life [3]. The LOS was defined as clinically suspected bacterial infection with onset of symptoms ≥ 5th day of life being either a blood culture positive sepsis (plausible pathogen) or a clinical sepsis in cases of negative cultures with elevated inflammatory markers (CRP [c-reactive protein] > 10 mg/L), and both had to be treated with antibiotics for at least 7 days. The IVH grades II and I were defined as mild hemorrhage and grades III and periventricular hemorrhagic infarction (PVHI, former IVH grade IV) as severe [10]. Data concerning the cause, localization, type (multiple vs. single) and day of diagnosis of the perforation were documented in every single case. Surgical, radiological and histological findings led to diagnosis of GIT perforations and the etiology. In addition, data concerning the follow-up and the neurodevelopmental outcome were collected at the corrected for prematurity age of 2 years at the outpatient clinic of neurodevelopmental follow-up. The neurodevelopmental outcome included death, age of death, the follow-up rate, the age of testing, normal development, cognitive or motor deficits, microcephaly, dystrophy, strabismus, seizures, behavioral disorders and visual or hearing impairment. For 2‑year outcome the Bayley scales of infant development second edition (BSID-II) test battery was used until 2015, and in 2016 the BSID III was introduced in our outpatient Clinic of Neurodevelopmental Outcome. Due to the small numbers a descriptive form of neurodevelopmental outcome was preferred instead of scores. The Bayley-III cognitive composite score was reported to be 7.1 points higher than the Bayley-II mental developmental index (MDI) and the Bayley-III motor composite score 8.4 points higher than the Bayley-II psychomotor developmental index (PDI) [11]. We took these differences in consideration and adapted the Bayley-III scores accordingly to get comparable and homogeneous results regarding normal outcome. Jary et al. recommended using cut-off threshold values for the BSID-III of 85 for moderate impairment, and 70 for severe impairment comparable with BSID-II threshold of 70 and 55 [12]. In our cohorts, only five cases and five controls were tested with the BSID-III.

### Data analysis

Statistical analyses were performed using Microsoft Excel© and IBM SPSS© statistics (Version 24.0. IBM Corp, Armonk, NY, USA). Descriptive statistics were done using mean and standard deviances for perinatal and neonatal data, median and range (due to the small sample number) for GIT perforation descriptions and for both numbers with percentages as adequate. For calculations of incidences, all preterm infants of 32 weeks (+6 days) were included with hospitalization at the neonatal wards during the study period. The Student’s *t*-test and Wilcoxon test for non-categorical data and the χ^2^-test using Yates correction and Fisher’s exact test for categorical data were used as appropriate. Statistical significance was set at *p* < 0.05.

## Results

### Incidence

**Fig. 1:**
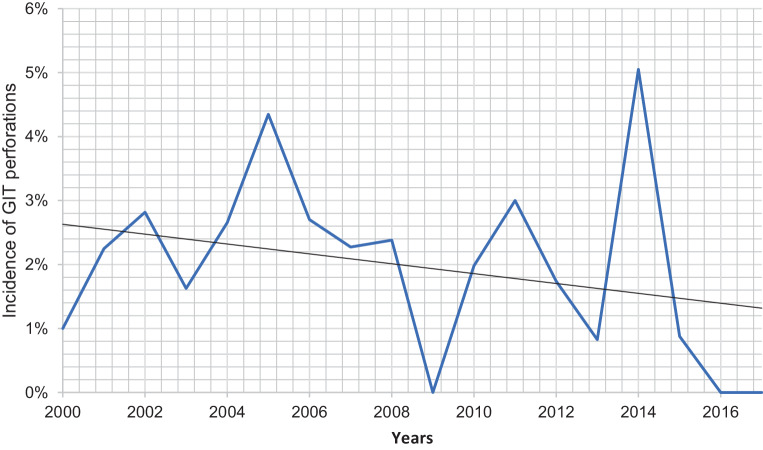
Incidence of gastrointestinal perforations among preterm infants’ ≤ 32 weeks of gestational age between 2000 and 2017 in Graz, Austria

During the period of study, 1878 very preterm infants (≤ 32 + 6 weeks) were treated at our NICU of the Medical University of Graz. Out of this population 38 infants (2.02%) had GIT perforation. Fig. 1 shows the incidence of GIT perforation during the study period. The highest incidence was 5.1% in 2014, the lowest was 0% in the years 2009, 2016 and 2017. There was a tendency to a decline of GIT perforations over the 18 years, but the trend was not significant (2000–2008 vs. 2009–2017, *p* = 0.08). Since 2010 we actively treat preterm infants with 23 weeks of gestational age.

### Perinatal and neonatal data

Perinatal and neonatal characteristics are shown in Table 1. Infants with perforations had a lower Apgar score at 1 min, higher rates of LOS, more days on mechanical ventilation, more severe RDS with the need for more surfactant doses and more severe ROP, and a longer duration of total hospitalization and hospitalization at the NICU. Rates of mild and severe IVH differed between groups (*p* = 0.037), but differences were no longer evident when neonatal deaths were excluded (*p* = 0.186), see Table 1.

**Table 1:** Perinatal and neonatal characteristics of 38 very preterm infants with gastrointestinal tract perforation and 76 matched controls between 2000 and 2017

Parameters	Cases (*n* = 38)	Controls (*n* = 76)	*p*-value
Gestational age (weeks)	26.3 ± 2.2	26.3 ± 2.1	Ns
Birth weight (g)	878 ± 313	894 ± 304	Ns
Gender (male: female)	24 (63): 14 (37)	47 (62): 29 (38)	Ns
Small for gestational age	8 (21)	10 (13)	Ns
Twins/triplets	14 (37)/0 (0)	22 (29)/1 (3)	Ns/ns
Maternal age (years)	30.1 ± 5.6	31.0 ± 6.4	Ns
Cesarean section	29 (76)	65 (86)	Ns
Chorioamnionitis	14 (37)	26 (34)	Ns
Apgar score at 1 min	6.6 ± 1.8	5.8 ± 2.3	0.040
Apgar score at 5 min	8.1 ± 1.3	7.9 ± 1.7	Ns
Umbilical artery pH	7.32 ± 0.07	7.29 ± 0.12	Ns
Length of stay (days)	122 (33–217^a^)	83 (20–168^b^)	< 0.001
Mechanical ventilation (days)	30 (6–125^a^)	18 (1–91^b^)	0.013
Early onset sepsis	8 (21)	11 (16)	Ns
RDS	29 (76)	64 (84)	Ns
RDS grading (1–4)	3.0 ± 1.0	2.3 ± 1.2	0.003
Surfactant	28 (73)	59 (76)	Ns
Doses/infant	1.3 ± 0.6	1.1 ± 0.3	0.002
Intraventricular hemorrhage Mild IVH Severe IVH/PVHI	13 (34) 11 (29) 2 (5.3)^c^	22 (29) 9 (12) 13 (17)^c^	Ns
PVE/cystic PVL	4 (11)/3 (8)	7 (9.2)/4 (5.3)	Ns/ns
Neonatal seizures	5 (13)	4 (5.3)	0.072
Late onset sepsis	21 (55)	16 (21)^c^	< 0.001
Bronchopulmonary dysplasia	4 (11)	14 (18)	Ns
ROP	7 (18)	11 (15)	Ns
ROP grading (1–5)	2.7 ± 1.1	0.5 ± 1.0	< 0.001

Data are given as mean ± SD; median (range) or number (%)

^a^Data calculated for survivors only (*n* = 34)

^b^Data calculated for survivors only (*n* = 65)

^c^Deaths 1 (cases) and 8 (controls)

*IVH* intraventricular hemorrhage, *PVHI* periventricular hemorrhagic infarction, *PVE* periventricular echodensities (*PVL* I), *PVL* periventricular leukomalacia, *ROP* retinopathy of prematurity, *RDS* respiratory distress syndrome, *STROBE* STrengthening the Reporting of OBservational studies in Epidemiology, *Ns* not significant

Small for gestational age = birth weight < 10. percentile

### Analysis of GIT perforations

The causes of GIT perforations (see Table 2) were (1) meconium obstruction of prematurity (delayed meconium passage with ileus and perforation) in 19 cases (50%) followed by (2) spontaneous intestinal perforation (SIP) in 7 cases (18%) and (3) necrotizing enterocolitis (NEC) in 6 cases (16%). There was one case of meconium ileus at day 1 of life with ileum perforation. Other causes included volvulus with perforation (*n* = 2/5%), and iatrogenic feeding tube (esophagus and stomach) and irrigation tube perforations (sigmoid colon; *n* = 3/8%).

**Table 2:** Localization of gastrointestinal tract perforations of 38 preterm infants ≤ 32 weeks of gestational age between 2000 and 2017

Localization of perforation	Cases (*n* = 38)	Causes
*Esophagus*	1 (3)	Iatrogenic
*Stomach*	1 (3)	Iatrogenic
*Small intestine*	31 (81)	–
Ileum, singular	27	MOP (*n* = 17), SIP (*n* = 6), NEC (*n* = 3), Meconium ileus (*n* = 1)
Ileum, multiple	3	NEC (*n* = 2), Volvulus (*n* = 1)
Jejunoileal passage	1	NEC
*Large intestine*	4 (10)	–
Appendix, singular	1	Volvulus
Left colic flexure, singular	1	MOP
Sigmoid colon, singular	2	Iatrogenic (*n* = 1), SIP (*n* = 1)
*Unknown*	1 (3)	MOP (death, no autopsy)

Data are given as *n* (%)

*MOP* meconium obstruction of prematurity, *SIP* spontaneous intestinal perforation, *NEC* necrotizing enterocolitis

**Fig. 2:**
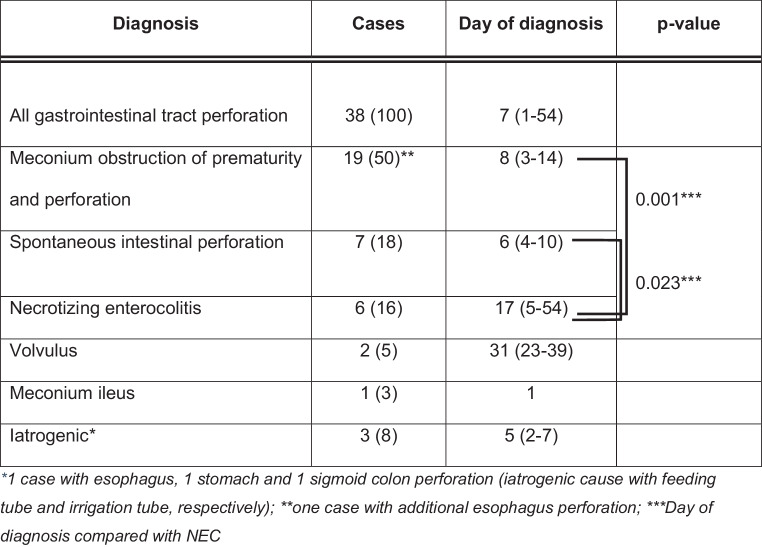
Diagnosis and time of gastrointestinal tract perforation in 38 preterm infants ≤ 32 weeks of gestational age between 2000 and 2018. Data are given as *n* (%) or median (range)

The majority of GIT perforations (*n* = 31/81%) were located in the small intestine, mainly singular in the Ileum (*n* = 27). Iatrogenic perforations of the (1) esophagus, (2) stomach, both by feeding tube, and (3) sigmoid colon by irrigation tube occurred all in extremely low gestational age infants. Time of GIT perforation is depicted in Fig. 2. Most of the perforations happened within the first 2 weeks of life. NEC associated perforation occurred significantly later compared to meconium obstruction of prematurity or SIP (*p* = 0.001 and 0.023, respectively).

### Mortality

Groups did not differ regarding mortality (11% vs. 17%; differences not significant). Infants with perforations died at a mean age of 15 days of life, controls at 22 days of life. Severe IVH and PVL were the most common causes of mortality (50% of deaths among cases with perforation, 73% of deaths among controls).

### Follow-up and outcome

Table 3 shows data on neurodevelopmental follow-up at 2 years of corrected age. Neurodevelopment follow-up did not differ between groups (normal development 42% vs. 51%). Cases had more often diagnoses of microcephaly and dystrophy at the age of 2 years (28% and 41% vs. 12% and 23%, *p* = 0.036 and 0.027, respectively). Subgroup analysis was done in cases with MOP, SIP and NEC, and rates of normal development were 42% (8/19), 43% (3/7), and 50% (3/6), respectively.

**Table 3:** Neurodevelopmental follow-up of 38 cases ≤ 32 weeks of gestational age with gastrointestinal tract perforations and 76 matched controls born at the age of 2 years (corrected for prematurity) between 2000 and 2017

Follow-up	Cases (*n* = 38)	Controls (*n* = 76)	*p*-value
Deaths	4 (11)	11 (17)	Ns
*Follow-up* at 2 years	33/34 (97)	65/65 (100)	Ns
Normal development	16 (49)	26 (40)	Ns
Cognitive/motor deficits	17(51)	39 (60)	Ns
Cognitive deficits	17 (51)	39 (60)	Ns
Development delay	14 (42)	33 (51)	Ns
Mental retardation	3 (9)	6 (9)	Ns
Motor deficits	2 (6)	7 (11)	Ns
Athetosis	1 (3)	3 (5)	Ns
Cerebral palsy	1 (3)	4 (6)	Ns
Microcephaly	9 (27)	8 (12)	0.036
Dystrophy	14 (42)	15 (23)	0.027
Visual impairment	4 (12)	9 (14)	Ns
Hearing impairment	0 (0)	0 (0)	Ns
Seizures	1 (3)	1 (1.5)	Ns
Behavioral disorders	4 (12)	4 (6)	Ns

Data are given as *n* (%)

*Ns* not significant

## Discussion

### Main findings

During the study period of 18 years we observed a low incidence of GIT perforation of 2% in very low birth weight infants. Main diagnoses were meconium obstruction of prematurity followed by SIP and NEC. Most perforations were singular and the main location was the terminal ileum. Mortality was not increased following GIT perforations and follow-up at the corrected for prematurity age of 2 years did not differ between groups. Because of higher neonatal morbidity cases more often had a diagnosis of dystrophy at the age of 2 years.

Shah et al. [13] and Kawase et al. [14] evaluated the incidence of GIT perforation in very low birthweight infants and reported rates of 2.4% [13] and 2.3% [14], respectively. Other studies stated higher rates of 5% [15] and 8.1% [16] in comparable patient groups. Similar to our findings, other studies demonstrated that particularly male infants [6, 15, 17, 18] and extremely preterm infants were affected by GIT perforations [13, 19, 20].

Intestinal obstruction due to delayed meconium passage presented the most common diagnosis for GIT perforation within our cases. The diagnosis of meconium obstruction was clearly defined and perforation was a diagnosis of excluding other causes. Although meconium obstruction of prematurity is increasingly recognized as a distinct clinical entity and risk factor for intestinal perforation [5], it has rarely been mentioned as a cause for intestinal perforation in other cohort studies [2, 13, 14, 21]. A key point to our opinion might be the fact that in close collaboration with neonatologists and pediatric surgeons we discuss cases with meconium obstruction of prematurity at a 6 h interval aiming to operate on them at the latest time point possible but before perforation and deterioration of the newborn by ileostomy. This is a process involving the most experienced consultants in the regular case discussion rounds. In general, the causes of GIT perforations differed widely between centers and studies [3, 4, 13, 14, 20, 21]; rates of NEC varied between 7.7% [14] and 58% [13], those of SIP between 6.3% [21] and 92.3% [14]. Thus, classifications of GIT perforations, especially perforations other than NEC associated ones [13], still seem to be a matter of debate and some standards would be greatly appreciated.

All studies agree that the small intestine and in particular the terminal ileum, presented the most common localization of GIT perforation [21, 22].

Infants with GIT perforation did not have a higher mortality rate than matched controls. The literature regarding GIT perforations and associated mortality differed widely. Eicher et al. [8] found no significant differences regarding mortality rates between infants with GIT perforation and matched controls among extremely low birth weight (ELBW) infants. Shah et al. [7] and Wadhawan et al. [6] found significantly higher mortality rates among ELBW infants with GIT perforations, but matching was not performed. Among our cases cerebral complications, mainly bleedings, were the most common causes of death and GIT perforations only played a minor role as cause of death. An early decompression by ileostomy might have been one factor reducing mortality rates independent of intestinal perforation or not, but this hypothesis remains highly speculative.

In accordance with Shah et al. [7] and Wadhawan et al. [6] infants with GIT perforations had a worse cognitive outcome beyond the 2‑year outcome. In contrast, Eicher et al. [8] found no differences between cases and matched controls. Larger studies (multicenter studies) are needed to probably better figure out the impact of GIT perforation on mortality and outcome of preterm infants. One point regarding similar neurodevelopmental outcome results might be the fact that most causes of GIT perforations had no associated systemic inflammatory response (MOP and SIP). Inflammation and cytokines are known to have detrimental effects on the premature brain [23]. Infants with GIT perforation had a more than doubled rate of LOS than controls with symptomatic inflammation that might have resulted in a worse outcome; however, there were no differences between groups.

### Strengths and limitations of the study

The long study period with high neurodevelopmental follow-up rates represented the strengths of this single-center study. The design of a careful retrospective matched case-control study according to the STROBE standards ensured high-quality comparisons between groups; however, the single center study design led to a relatively small number of cases; a general problem regarding rare entities/complications of neonatal intensive care. Many cases with perforations might not have had an inflammatory response syndrome, thus, despite higher rates of LOS, this might add to the generally acceptable outcome without differences between groups.

## Conclusion

Despite increased morbidities and longer neonatal hospitalization preterm infants with GIT perforation had neither increased mortality nor a worse neurodevelopmental outcome at the age of 2 years.
